# Association between TV/DVD screen exposure time at age 1 and risk of chronic constipation at age 3: the Japan Environment and Children’s Study

**DOI:** 10.1265/ehpm.25-00109

**Published:** 2025-10-16

**Authors:** Masashi Hotta, Satoyo Ikehara, Makiko Tachibana, Kazuko Wada, Junji Miyazaki, Tadashi Kimura, Ryo Kawasaki, Hiroyasu Iso, The Japan Environment and Children’s Study Group

**Affiliations:** 1 Osaka Regional Center for Japan Environment and Children’s Study (JECS), The University of Osaka, Suita, Osaka, Japan; 2 Laboratory of Vaccine Materials/Laboratory of Gut Environmental System, Microbial Research Center for Health and Medicine, National Institutes of Biomedical Innovation, Health and Nutrition (NIBN), Ibaraki, Osaka, Japan; 3 Department of Neonatal Medicine, Osaka Women’s and Children’s Hospital, Izumi, Osaka, Japan; 4 Department of Epidemiology and Behavioral Science, Faculty of Medicine, University of the Ryukyus, Ginowan, Okinawa, Japan; 5 Department of Pediatrics, Graduate School of Medicine, The University of Osaka, Suita, Osaka, Japan; 6 Public Health, Department of Social and Environmental Medicine, Graduate School of Medicine, The University of Osaka, Suita, Osaka, Japan; 7 Sakai City Hospital Organization, Sakai, Osaka, Japan; 8 Institute for Global Health Policy Research, Bureau of International Health Cooperation, National Center for Global Health and Medicine, Shinjuku, Tokyo, Japan

**Keywords:** Infant, Birth cohort, Constipation, Screen time

## Abstract

**Background:**

Chronic constipation is a long-term problem that decreases children’s quality of life. Information and communication technology devices have developed rapidly in recent decades and have had various impacts on children. This prospective cohort study examined the association between television/digital versatile disc (TV/DVD) screen exposure time at age 1 and the risk of chronic constipation at age 3.

**Methods:**

Data from 63,697 infants in the Japan Environment and Children’s Study (JECS) were analyzed. We divided participants into five groups according to TV/DVD exposure time per day: no exposure (0 h), short exposure (<1 h), middle exposure (1.0–<2.0 h), long exposure (2.0–<4.0 h), and very long exposure (≥4 h). Logistic regression analysis was performed to assess the association between TV/DVD exposure time and the risk of constipation. For logistic regression analysis, odds ratios (ORs) were adjusted for sex, parents’ education, household income, nursery school, feeding contents, and obesity. The interaction between the sexes was also examined.

**Results:**

The prevalence of constipation for males, females, and all participants at age 3 was 9.3, 11.0, and 10.1%, respectively. The TV/DVD screen time distribution per day at age 1 was 10.6% for none, 34.1% for short, 29.9% for middle, 19.2% for long, and 6.2% for the very long exposure group. After adjusting for confounding factors, a dose-response pattern was identified between TV/DVD exposure time and constipation in all participants (p for trend < 0.001). The adjusted ORs increased progressively in the short (OR 1.15, 95% confidence interval [CI] 1.04–1.27), middle (OR 1.22, 95% CI 1.11–1.35), long (OR 1.37, 95% CI 1.24–1.52), and very long exposure groups (OR 1.53, 95% CI 1.35–1.74). This association was not significantly different between the sexes (p for interaction = 0.36).

**Conclusions:**

Longer TV/DVD exposure time at age 1 was associated with the risk of chronic constipation at age 3. Excessive screen exposure may need to be avoided from infancy to decrease the risk of chronic constipation in later years.

**Supplementary information:**

The online version contains supplementary material available at https://doi.org/10.1265/ehpm.25-00109.

## Introduction

Constipation in children is common, with a frequency of 0.5–32.2% [1]. Chronic constipation is a long-term problem that decreases the quality of life of children, and early and appropriate evaluation and intervention are usually beneficial [2–5]. Constipation in children is most common during three periods: during the transition from breast milk to artificial milk or weaning in infants, during toilet training in toddlers, and at the start of school or when children avoid defecation at school [6]. Of these three periods, the peak onset of constipation is during toilet training at 2–4 years [1, 7]. More than 40% of children diagnosed with constipation at age 4 or younger still have symptoms of constipation at school age despite treatment [8]. Additionally, constipation in children is associated with a higher risk of later psychological symptoms and mental disorders [1, 5] though appropriate treatment of constipation can improve quality of life [5]. Therefore, early diagnosis and appropriate treatment are essential.

Information and communication technology devices such as televisions (TV), digital versatile discs (DVDs), computers, and mobile terminals have developed rapidly in recent decades and have had various impacts on children. Particularly, their incorporation into education and learning has reportedly increased learning opportunities and motivated students [9]. However, there are also reports of increased risk of headaches, musculoskeletal and visual abnormalities, and internet addiction [9, 10].

Screen time is considered a common sedentary behavior in children and adults [11, 12], and reduced screen time may lead to increased physical activity [12]. Previous studies revealed that electronic screen time of >2 h was associated with two times more probability of constipation in Bangladesh school-aged children (odds ratio [OR] 2.138, 95% confidence interval [CI] 1.063–4.301) [13] and more than 2 h of recreational screen time was associated with chronic constipation in Chinese high school students (OR 1.583, 95% CI 1.351–1.847) [14]. Another study showed the correlation between screen time for television/computer and the occurrence of chronic functional constipation in children aged 4–18 years (F = 92.161, p < 0.001) [15]. However, the sample size of these studies was too small to determine the association, and no study has prospectively examined the association between screen time and the risk of constipation in children, especially at 2–4 years, when constipation is most likely to occur.

In this study, we used large nationwide birth cohort data to investigate the association between screen time at age 1 and constipation at age 3.

## Methods

### Study design and participants

This longitudinal study used the Japan Environment and Children’s Study (JECS) data. The JECS is a nationwide birth cohort study in Japan funded by the Ministry of the Environment. Details of participant enrollment, inclusion criteria, and the JECS study design have been described previously [16, 17]. Briefly, we recruited pregnant women as early as possible from cooperating healthcare providers, including hospitals and clinics within or near the study areas, in response to 15 Regional Centers’ requests for cooperation. The Regional Centers are located in universities throughout Japan. We registered 104,062 fetal records between January 2011 and March 2014. Outcome, exposure, and covariate data were obtained through self-administered questionnaires and medical record transcriptions. In the JECS, the questionnaires were distributed at enrollment, during the second or third trimester of pregnancy, 1 month after childbirth, and every 6 months after childbirth until 3 years of age. Additionally, medical records at enrollment, delivery, and 1 month after birth were transcribed by physicians, midwives/nurses, and/or Research Co-ordinators. This study was conducted according to the principles of the Declaration of Helsinki. Written informed consent was obtained from the infants’ parents/legal guardians/next of kin. The JECS protocol was reviewed and approved by the Ministry of the Environment’s Institutional Review Board for Epidemiological Studies and the Ethics Committees of all participating institutions (Ethical Number: No.100910001).

**Fig. 1:**
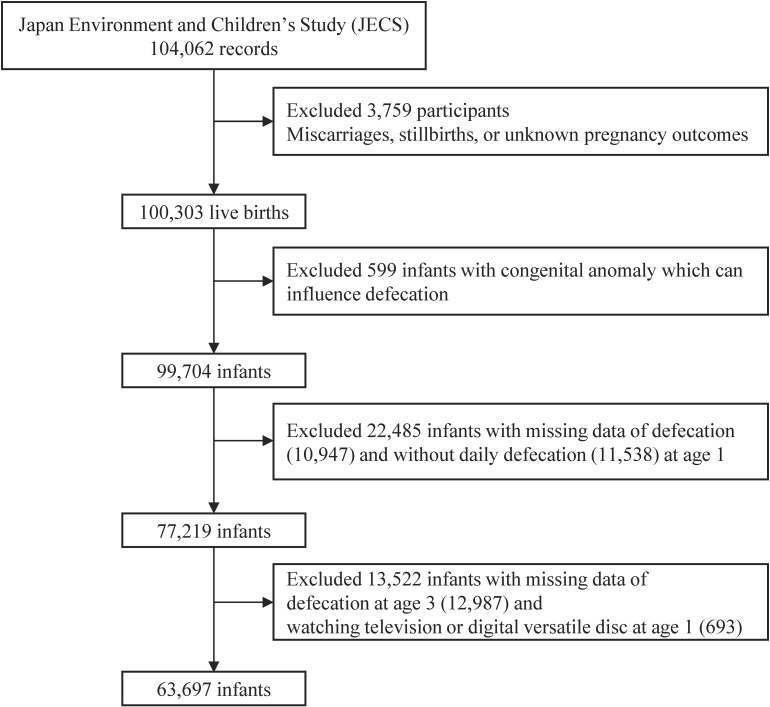
Flowchart of the study participants

This study was based on the jecs-ta-20190930 dataset released in October 2019 and revised in February 2020. From the fixed JECS data, we excluded 3,759 cases with miscarriages, stillbirths, and unknown pregnancy outcomes. We also excluded 599 infants with congenital anomalies that could influence defecation. Congenital anomalies that can influence defecation include chromosomal abnormalities, anencephaly, hydrocephalus, holoprosencephaly, congenital diaphragmatic hernia, omphalocele, gastroschisis, esophageal atresia, duodenal atresia, small bowel atresia, imperforate anus, and meningocele. Additionally, we excluded 22,485 infants with missing data on defecation and without daily defecation at age 1 and 13,522 infants with missing data on defecation at age 3 and watching TV/DVD at age 1. The remaining 63,697 infants were included in this study (Fig. 1). The selected characteristics of parents and children did not differ from those of the JECS population (Supplementary Table 1).

### Exposure measures

The exposure was the children’s TV/DVD screen time per day at age 1. In the questionnaire, we asked parents or guardians about their children’s TV/DVD screen time at age 1: “How many hours do you let your children watch TV or DVD on a typical day?” The responses were obtained from five categories: none, <1 h, 1.0–<2.0 h, 2.0–<4.0 h, and ≥4.0 h.

### Outcome measure

The primary outcome was the prevalence of chronic constipation at age 3. We used the ROME III criteria [6, 18] at age 3 to measure constipation. Briefly, chronic constipation was defined as having more than 1 month of at least two of the following: two or fewer defecations per week, at least one episode per week of incontinence after the acquisition of toileting skills, history of excessive stool retention, history of painful or hard bowel movements, presence of a large fecal mass in the rectum, and history of large-diameter stools that may obstruct the toilet. This information was obtained from a questionnaire at age 3; ROME IV criteria could not be used because the questionnaire at age 3 was developed before the recruitment and did not include the items used in the ROME IV criteria.

### Statistical analysis

We calculated the prevalence of potential confounders according to the five categories of TV/DVD exposure at age 1. Logistic regression analysis was used to adjust for potential risk factors and assess the association between TV/DVD duration at age 1 and the risk of constipation at age 3. For the logistic regression analysis, ORs were adjusted for the following confounding factors: mother’s tertiary education (yes, no, and missing), father’s tertiary education (yes, no, and missing), household income (<4 million Japanese yen (JPY), 4≤ and <6 million JPY, ≥6 million JPY, and missing), and nursery school (yes, no, and missing), feeding contents (mother’s own milk only, mixed, artificial milk only, and missing), obesity [19] (yes, no, and missing), and sex (male/female). Obesity was defined as the degree of obesity in this cohort (body weight/mean body weight of included infants-1) ≥ 20% [20]. Data on the parents’ education and household income were obtained from the questionnaires during the second or third trimesters of pregnancy. We used “missing” for each variable as a category and analyzed it in the model. We examined the interaction between sexes because the effect of sex on the risk of constipation is inconsistent [1, 21]. After logistic regression analysis, a trend analysis was performed by allocating 0, 1, 2, 3, and 4 for the no, short, middle, long, and very long exposure groups for ORs, respectively. Statistical significance was set at p-value < 0.05. All statistical analyses were performed using the R software (R Foundation for Statistical Computing, Vienna, Austria, version 4.2.1).

## Results

The baseline characteristics of the participants are presented in Table 1. The prevalence of constipation for males, females, and all participants at age 3 was 9.3, 11.0, and 10.1%, respectively. The TV/DVD screen time distribution per day at age 1 was 10.6% for none, 34.1% for <1 h, 29.9% for 1.0–<2.0 h, 19.2% for 2.0–<4.0 h, and 6.2% for ≥4.0 h. Infants with longer TV/DVD exposure times were less likely to be male, had lower parental education and household income, had a higher proportion of no attendance at nursery school, and were more likely to consume artificial milk than those with shorter TV/DVD exposure times.

**Table 1:** Participants’ characteristics

	**Total**	**TV/DVD exposure**				

		**No**	**Short**	**Middle**	**Long**	**Very long**
No. of participants	63697	6766	21737	19019	12239	3936
Constipation (%)	6427 (10.1)	565 (8.3)	2070 (9.5)	1920 (10.1)	1376 (11.2)	496 (12.6)
Male (%)	32812 (52)	3628 (54)	11313 (52)	9661 (51)	6195 (51)	2015 (51)
Males with constipation (%)	3040 (9.3)	268 (7.4)	1038 (9.2)	865 (9.0)	641 (10.3)	228 (11.3)
Females with constipation (%)	3389 (11.0)	297 (9.5)	1032 (9.9)	1055 (11.3)	737 (12.2)	268 (14.0)
Mother’s tertiary education (%)						
Yes	42213 (66)	4859 (72)	14917 (69)	12676 (67)	7590 (62)	2171 (55)
No	20873 (33)	1838 (27)	6625 (31)	6159 (32)	4531 (37)	1720 (44)
Missing	611 (1.0)	69 (1.0)	195 (0.9)	184 (1.0)	118 (1.0)	45 (1.1)
Father’s tertiary education (%)						
Yes	36778 (58)	4098 (61)	12635 (58)	11156 (59)	6852 (56)	2037 (52)
No	26011 (41)	2571 (38)	8820 (41)	7597 (40)	5197 (43)	1826 (46)
Missing	908 (1.4)	97 (1.4)	282 (1.3)	266 (1.4)	190 (1.6)	73 (1.9)
Household income (%)						
<4 million JPY	22644 (36)	2253 (33)	7140 (33)	6716 (35)	4795 (39)	1740 (44)
4≤, <6 million JPY	20080 (32)	2023 (30)	6998 (32)	6102 (32)	3826 (31)	1131 (29)
≥6 million JPY	16718 (26)	2065 (31)	6260 (29)	4938 (26)	2698 (22)	757 (19)
Missing	4255 (6.7)	425 (6.3)	1339 (6.2)	1263 (6.6)	920 (7.5)	308 (7.8)
Nursery school at age 1 (%)						
Yes	17718 (28)	2748 (41)	7499 (35)	4771 (25)	2260 (19)	440 (11)
No	45737 (72)	3986 (59)	14148 (65)	14172 (75)	9941 (81)	3490 (89)
Missing	242 (0.4)	32 (0.5)	90 (0.4)	76 (0.4)	38 (0.3)	6 (0.2)
Feeding contents at age 1 (%)						
Mother’s milk only	25664 (40)	2774 (41)	8835 (41)	7708 (41)	4908 (40)	1439 (37)
Mixed	9201 (14)	1087 (16)	3467 (16)	2646 (14)	1534 (13)	467 (12)
Artificial milk only	19350 (30)	1903 (28)	6380 (29)	5798 (31)	3897 (32)	1372 (35)
Missing	9482 (15)	1002 (15)	3055 (14)	2867 (15)	1900 (16)	658 (17)
Obesity at age 1 (%)						
Yes	1672 (2.6)	119 (1.8)	550 (2.5)	522 (2.7)	366 (3.0)	115 (2.9)
No	46222 (73)	4695 (69)	15500 (71)	14002 (74)	9110 (74)	2915 (74)
Missing	15803 (25)	1952 (29)	5687 (26)	4495 (24)	2763 (23)	906 (23)

JPY, Japanese yen; TV/DVD, television/digital versatile disc

Table 2 presents the ORs for constipation risk according to TV/DVD exposure at age 1. The TV/DVD exposure time was positively associated with the risk of constipation at age 3. After adjusting for confounding factors, a dose-response pattern was found between TV/DVD exposure time and the risk of constipation in all participants and in both sexes (p for trend < 0.001). The adjusted ORs increased progressively in the short (OR 1.15, 95% CI 1.04–1.27), middle (OR 1.22, 95% CI 1.11–1.35), long (OR 1.37, 95% CI 1.24–1.52), and very long exposure groups (OR 1.53, 95% CI 1.35–1.74) in all participants. There was no sex difference in this association (p for interaction = 0.36).

**Table 2:** Odds ratio for the risk of chronic constipation at age 3 according to TV/DVD exposure time at age 1

	**TV/DVD exposure**					**p for trend**

	**No**	**Short**	**Middle**	**Long**	**Very long**	
Total participants	6766	21737	19019	12239	3936	
Cases (%)	565 (8.3)	2070 (9.5)	1920 (10.1)	1376 (11.2)	496 (12.6)	
Crude OR (95% CI)	1.00	1.16 (1.05–1.27)	1.23 (1.12–1.36)	1.39 (1.26–1.54)	1.58 (1.39–1.80)	<0.001
Adjusted OR (95% CI)^a^	1.00	1.15 (1.04–1.27)	1.22 (1.11–1.35)	1.37 (1.24–1.52)	1.53 (1.35–1.74)	<0.001

Male	3628	11313	9961	6195	2015	
Cases (%)	268 (7.4)	1038 (9.2)	865 (9.0)	641 (10.3)	228 (11.3)	
Crude OR (95% CI)	1.00	1.27 (1.10–1.46)	1.23 (1.07–1.42)	1.45 (1.25–1.68)	1.60 (1.33–1.93)	<0.001
Adjusted OR (95% CI)^b^	1.00	1.26 (1.10–1.45)	1.23 (1.07–1.43)	1.44 (1.24–1.68)	1.57 (1.30–1.90)	<0.001

Female	3138	10424	9358	6044	1921	
Cases (%)	297 (9.5)	1032 (9.9)	1055 (11.3)	737 (12.2)	268 (14.0)	
Crude OR (95%CI)	1.00	1.05 (0.92–1.20)	1.22 (1.06–1.39)	1.33 (1.15–1.53)	1.55 (1.30–1.85)	<0.001
Adjusted OR (95% CI)^b^	1.00	1.04 (0.91–1.20)	1.20 (1.05–1.38)	1.30 (1.13–1.51)	1.49 (1.25–1.78)	<0.001

^a^Adjusted for mother’s tertiary education, father’s tertiary education, household income, nursery school at age 1, feeding contents at age 1, obesity at age 1, and sex.

^b^Adjusted for mother’s tertiary education, father’s tertiary education, household income, nursery school at age 1, feeding contents at age 1, and obesity at age 1.

TV/DVD, television/digital versatile disc; OR, odds ratio; CI, confidence interval

## Discussion

This study revealed the association between TV/DVD exposure time at age 1 and constipation at age 3. Longer TV/DVD exposure at age 1 was associated with a higher risk of constipation at age 3 in a dose-dependent manner. To our knowledge, this is the first prospective cohort study to show this association.

The prevalence of constipation is approximately between 0.5% and 32.2% [1]. In our study, the prevalence was 10.1%, which is within the above range obtained from prior research findings. With a large sample size and adjustment for potential confounding factors, our findings are more robust than the results from previous studies [13–15, 22]. Our study can be considered clinically more important because of its larger sample size, adjustment for potential confounders, and investigation of children at age 3 when constipation is likely to occur.

**Fig. 2:**
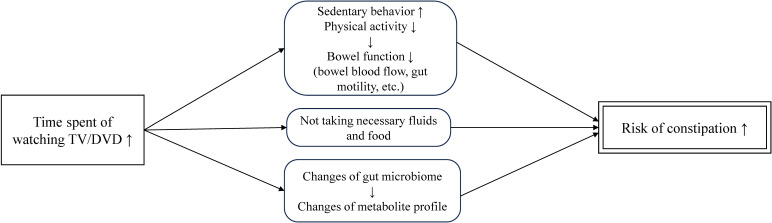
Potential pathways for the association between TV/DVD exposure and constipation. TV/DVD, television/digital versatile disc

Although the mechanisms underlying the association between TV/DVD exposure and chronic constipation remain unclear, several potential pathways exist (Fig. 2). First, longer exposure to TV/DVD may cause increased sedentary behavior and low physical activity in children [11, 12, 23]. Previous reports revealed that though colonic phasic activity decreased in the acute phase of exercise, colonic propulsion was enhanced during postexercise periods [24]. Repetitive exercises improve bowel blood flow, neuro-immuno-endocrine systems, gastrointestinal motility, mechanical bouncing of the gut, and fiber intake due to increased energy expenditure, which may positively impact bowel function [25]. Sedentary behavior may not provide such benefits. Second, prolonged exposure to TV/DVD can lead to constipation. By concentrating on watching TV/DVD for a long time, children may not take in the necessary amounts of fluids and food, which may lead to constipation. Third, screen time may also be associated with the gut environment. Mice with voluntary wheel running had more fecal *Bifidobacterium* than sedentary mice [26] though the result of animal experiments may not necessarily be true for humans. Increased fecal *Bifidobacterium* was associated with increased stool frequency [27] and improved bowel function [28, 29] in adults with constipation. Additionally, longer screen time was associated with higher percentages of the genera *Prevotella*, *Veillonella*, *Bacteroides*, *Lachnospira*, *Coprococcus*, and *Ruminococcus*, and metabolome profiles of lower concentrations of proline, alanine, 1-methylhistidine, picolinic acid, and tyrosine in fecal samples of college students [30]. Although the mechanisms underlying the association between constipation and the gut environment have not yet been elucidated, changes in the gut environment due to prolonged screen time may cause constipation in children.

Based on the findings of our study, excessive exposure to TV/DVD may need to be avoided to reduce the risk of chronic constipation. While several studies examined the association between screen exposure and other health problems in children [9, 10], it is essential that public health experts and policymakers fully understand these findings and work to disseminate this information to the general public, including pregnant women and families with young children. Additionally, screen time in early childhood is influenced by social factors such as parental mental health and the availability of social support [31]. Therefore, strengthening social support for parents raising children is important.

The strengths of this study include its large, nationwide, prospective cohort design and use of potential confounding factors for adjustment. Additionally, we detected a dose-response relationship between TV and DVD exposure time at age 1 and chronic constipation at age 3, when constipation was most likely to occur [7].

However, this study had some limitations. First, we did not obtain information on family history of constipation [21], parents’ screen time, infant diets (e.g., insufficient fiber intake) [32, 33], and physical activities which may be potential confounding factors. Therefore, there remains a possibility of residual confounding. However, especially in terms of lifestyle such as diets and physical activities, we adjusted for related variables such as parental education, household income, nursery school, feeding contents, and obesity, which may indirectly reflect differences in lifestyle. Future studies should aim to more precisely measure and adjust for lifestyle-related variables. Second, we did not assess when children began to be exposed to the screens. The total daily exposure time and the time of screen exposure initiation may be related to constipation. Additionally, though many children are exposed to portable electronic devices such as tablets and cellular phones, we did not evaluate the exposure to these devices because our questionnaire at age 1 did not include this information. Third, this observational study did not demonstrate any causal relationships. Fourth, the generalizability of our findings to other races and ages remains uncertain because of the characteristics of the participants. Further studies in other countries and regions, including children of other ages, are required.

## Conclusion

Longer TV/DVD time spent at age 1 was positively associated with the risk of chronic constipation at age 3 in a dose-dependent manner. Excessive exposure to screens may need to be avoided in infants to decrease the risk of chronic constipation in later years.

## Abbreviations

CI: confidence interval
DVD: digital versatile disc
JECS: Japan Environment and Children’s Study
JPY: Japanese yen
OR: odds ratio
TV: television

## Supplementary information

**Additional file 1:** **Supplementary Table 1.** Comparison between included and excluded infants.

## Declarations

### Availability of data and material

Data are unsuitable for public deposition due to ethical restrictions and the legal framework of Japan. It is prohibited by the Act on the Protection of Personal Information (Act No. 57 of May 30, 2003, amendment on September 9, 2015) to publicly deposit data containing personal information. Ethical Guidelines for Medical and Health Research Involving Human Subjects, enforced by the Japan Ministry of Education, Culture, Sports, Science and Technology and the Ministry of Health, Labor and Welfare, also restrict the open sharing of epidemiologic data. All inquiries about access to data should be sent to jecs-en@nies.go.jp. The person responsible for handling inquiries sent to this e-mail address is Dr. Shoji F. Nakayama, JECS Programme Office, National Institute for Environmental Studies.
