# Supplementary material for: Association between TV/DVD screen exposure time at age 1 and risk of chronic constipation at age 3: the Japan Environment and Children’s Study
Source: Environ Health Prev Med. 2025 Oct 16;30:80. doi: 10.1265/ehpm.25-00109 (PMC12550413; doi:10.1265/ehpm.25-00109)
Supplement: Supplementary file 1 — Additional file 1: Supplementary Table 1. Comparison between included and excluded infants. [file ehpm-30-080-s001.docx]

Supplementary Table 1. Comparison between included and excluded infants

|  | Total infants | Included infants | Excluded infants |
| --- | --- | --- | --- |
| No. of participants | 77219 | 63697 | 13522 |
| Mother’s tertiary education (%) |  |  |  |
| Yes | 49829 (65) | 42213 (66) | 7616 (56) |
| No | 26468 (34) | 20873 (33) | 5595 (41) |
| Missing | 922 (1.2) | 611 (1.0) | 311 (2.3) |
| Father’s tertiary education (%) |  |  |  |
| Yes | 43363 (56) | 36778 (58) | 6585 (49) |
| No | 32503 (42) | 26011 (41) | 6492 (48) |
| Missing | 1353 (1.8) | 908 (1.4) | 445 (3.3) |
| Household income (%) |  |  |  |
| < 4 million JPY | 27799 (36) | 22644 (36) | 5155 (38) |
| 4 ≤, < 6 million JPY | 23932 (31) | 20080 (32) | 3852 (29) |
| ≥ 6 million JPY | 19746 (26) | 16718 (26) | 3028 (22) |
| Missing | 5742 (7.4) | 4255 (6.7) | 1487 (11) |
| Nursery school at age 1 (%) |  |  |  |
| Yes | 21652 (28) | 17718 (28) | 3934 (29) |
| No | 55246 (72) | 45737 (72) | 9509 (70) |
| Missing | 321 (0.4) | 242 (0.4) | 79 (0.6) |
| Feeding contents at age 1 (%) |  |  |  |
| Mother’s own milk only | 30471 (40) | 25664 (40) | 4807 (36) |
| Mixed | 10990 (14) | 9201 (14) | 1789 (13) |
| Artificial milk only | 24359 (32) | 19350 (30) | 5009 (37) |
| Missing | 11399 (15) | 9482 (16) | 1917 (14) |
| Obesity at age 1 (%) |  |  |  |
| Yes | 2036 (2.6) | 1672 (2.6) | 364 (2.7) |
| No | 55638 (72) | 46222 (73) | 9416 (70) |
| Missing | 19545 (25) | 15803 (25) | 3742 (28) |
| Sex (%) |  |  |  |
| Male | 40039 (52) | 32812 (52) | 7227 (53) |
| Female | 37178 (48) | 30885 (48) | 6293 (47) |
| Missing | 2 (0.0) | 0 (0.0) | 2 (0.0) |

JPY, Japanese yen
